# Stable nutritional endosymbiosis across cryptic diversity of a leafhopper species complex

**DOI:** 10.1186/s12864-026-12986-3

**Published:** 2026-05-25

**Authors:** Anna Michalik, Emilia Majewska, Veronika Andriienko, Karol H. Nowak, Adam Stroiński, Piotr Łukasik

**Affiliations:** 1 Department of Developmental Biology and Invertebrate Morphology, Institute of Zoology and Biomedical Research, Faculty of Biology, Jagiellonian University, Kraków, Poland; 2 Institute of Environmental Sciences, Faculty of Biology, Jagiellonian University, Kraków, Poland; 3 Doctoral School of Exact and Natural Sciences, Jagiellonian University, Kraków, Poland; 4 Museum and Institute of Zoology, Polish Academy of Sciences, Warsaw, Poland

**Keywords:** Auchenorrhyncha, Hemiptera, Cicadellidae, *Diplocolenus*, Leafhoppers, Amplicon sequencing, Metabarcoding, Metagenomics, Endosymbiosis, Bacteriome

## Abstract

**Background:**

Ancient nutritional symbioses underpin the ecological success of many sap-feeding insects. In ‘true hoppers’ - the hemipteran suborder Auchenorrhyncha, obligate bacterial partners provide essential amino acids lacking in plant phloem diets. However, the stability and persistence of such associations across the diversity of hoppers are poorly understood, and investigations are often complicated by insufficiently resolved host identity.

**Results:**

Here, we combined multitarget amplicon sequencing, metagenomics, and microscopy to assess the compositional and functional diversity of the microbiota across Polish, Swedish, and Austrian populations of leafhoppers morphologically identified as *Verdanus abdominalis*. Host COI data revealed pronounced cryptic genetic diversity, indicating several deeply divergent lineages within the characterized collection, but limited microbiota variation among populations. 16S rRNA amplicon data confirmed the consistent presence of the ancient bacterial endosymbionts *Candidatus* Sulcia muelleri and *Candidatus* Nasuia deltocephalinicola, and metagenomics showed that their reduced but complementary genomes jointly encode the complete set of essential amino acid biosynthesis pathways required by the host. Other microbes were uncommon in these symbioses. Microscopy corroborated these findings, revealing conserved bacteriome organization and spatial separation of *Sulcia* and *Nasuia* within distinct bacteriocytes.

**Conclusions:**

Our results demonstrate that the *Sulcia-Nasuia* dual symbiosis remains evolutionarily stable across cryptic *Verdanus* diversity, underscoring the robustness of ancient nutritional partnerships despite ongoing host diversification.

**Supplementary Information:**

The online version contains supplementary material available at 10.1186/s12864-026-12986-3.

## Introduction

Microbial symbioses are fundamental to the evolution of animals, profoundly shaping their ecological success and diversification [1, 2]. Among the most consequential are heritable nutritional associations, which provide hosts with the metabolic capacity to exploit nutrient-poor diets such as plant sap or blood [3, 4]. Sap-feeding hemipteran insects provide a particularly clear example of how ancient intracellular nutritional symbionts have become indispensable partners, supplying essential amino acids and vitamins that are absent from phloem or xylem diets [5–8]. These symbioses represent some of the oldest and most stable mutualisms known, underpinning major adaptive radiations within the order. Beyond their nutritional functions, different functional categories of microbial symbionts also influence host reproduction, defense, and diverse other traits, illustrating multifaceted contributions to insect fitness and evolution [9, 10].

Within Hemiptera, the suborder Auchenorrhyncha (planthoppers, leafhoppers, treehoppers, spittlebugs, and cicadas) provides a model framework for investigating the evolutionary stability of heritable symbioses. Most of these “true hoppers” maintain ancient obligate bacterial partners that have co-diversified with their hosts for hundreds of millions of years [6, 8, 11–13]. Their primary symbiont, *Candidatus* Sulcia muelleri (thereafter: *Sulcia*), dates back roughly 300 million years and is consistently retained across host lineages [6, 8, 14, 15]. It is accompanied by lineage-specific partners. For example, leafhoppers and treehoppers (superfamily Membracoidea) also harbor *Candidatus* Nasuia deltocephalinicola (thereafter: *Nasuia*), which encodes the biosynthesis pathways for histidine and methionine – two amino acids that *Sulcia* cannot produce. Together, these two co-symbionts form an integrated metabolic consortium that collectively synthesizes all ten essential amino acids required by the host [12, 13, 16, 17]. These ancient co-symbionts exhibit extreme genome reduction, with gene counts approaching theoretical limits for cellular autonomy, and show long-term co-diversification with their insect hosts [3, 12, 18, 19]. Yet despite this ancient persistence, dual symbioses vary in stability across Auchenorrhyncha clades. In many lineages, the ancestral dual partnership has remained intact – but it is often complemented by further microbes, often providing vitamins [20–22]. Further, in numerous cases, one or both symbionts have been functionally replaced by bacteria such as *Sodalis*, or by Hypocreales fungi [8, 23, 24]. Such diversity of outcomes provides an exceptional model for studying the mechanisms and evolutionary constraints governing symbiont maintenance, replacement, or metabolic complementation, enabling exploration of how these processes unfold across host lineages of different ages and ecological niches.

Nevertheless, systematic sequencing-based investigations of leafhopper symbioses remain restricted to a few model species, such as *Macrosteles quadrilineatus* [12, 25] and *Nephotettix cincticeps* [26]. These systems have yielded foundational insights into the biology of obligate symbioses, yet they represent only a narrow phylogenetic and ecological subset of Cicadellidae. The vast diversity of non-model leafhoppers – comprising thousands of species distributed across diverse habitats, often ecologically and economically significant – remains poorly characterized with respect to their microbial associations. Consequently, we know little about how ancient endosymbionts, facultative bacteria, and other microbial partners are distributed and vary in function across the broader taxonomic spectrum of hosts [15, 27–29]. This gap extends across hierarchical levels: between subfamilies and tribes, but also among and within nominal species. A further complication is that the broader taxonomic framework of many leafhopper groups is unresolved, with considerable cryptic diversity likely concealed within morphologically defined species. Together, these limitations hinder our ability to evaluate how ancient or more recently acquired endosymbioses persist, diversify, or adapt within closely related insect hosts.

The leafhopper *Verdanus abdominalis* (Fabricius, 1803), also known as *Diplocolenus abdominalis* (https://www.gbif.org/species/4483583), represents a promising system to address these questions. This grass-feeding deltocephaline species is widely distributed across northern Europe and is frequently collected from meadows and grasslands. Although *Verdanus* has been included in faunistic surveys from Sweden and Poland, its population structure and genetic diversity remain unresolved [15, 30, 31]. Moreover, no genomic or microbiome analyses have yet been conducted for this genus. Our preliminary barcoding and multitarget amplicon data revealed substantial mitochondrial divergence within and among populations in Poland and Sweden, suggesting the presence of cryptic lineages within what is currently classified as *V*. *abdominalis*. This discovery raised questions about the relationship between geography, host genetic structure, microbial community composition, and these microbes’ functions. The *Verdanus* system thus provides a natural laboratory to examine the stability of ancient endosymbioses in the context of host diversification, offering an opportunity to compare microbial community profiles, endosymbiont genomic features, and bacteriome architecture across geographically and genetically distinct populations within a single nominal species.

The goal of this study was to comprehensively characterize the symbiotic associations of *Verdanus* leafhoppers from Sweden, Poland and Austria using complementary genomic and microscopic approaches. Specifically, we aimed (1) to assess host genetic diversity and identify potential cryptic lineages within populations morphologically assigned to *V. abdominalis*; (2) to determine bacterial and fungal community composition, and the factors shaping its variation across populations; and (3) to test for conservation of genomic organization, function, and tissue distribution of the ancient *Sulcia–Nasuia* endosymbiosis, as well as the potential roles of complementary microbial partners. We addressed these aims by integrating multitarget amplicon sequencing, metagenomic reconstruction, and microscopy across 147 specimens from Poland, Sweden, and Austria. Through this multi-omics framework, we provide new insights into the stability and flexibility of ancient nutritional symbioses within cryptically diversified insect hosts.

## Methods

### Specimen collection and identification

**Fig. 1:**
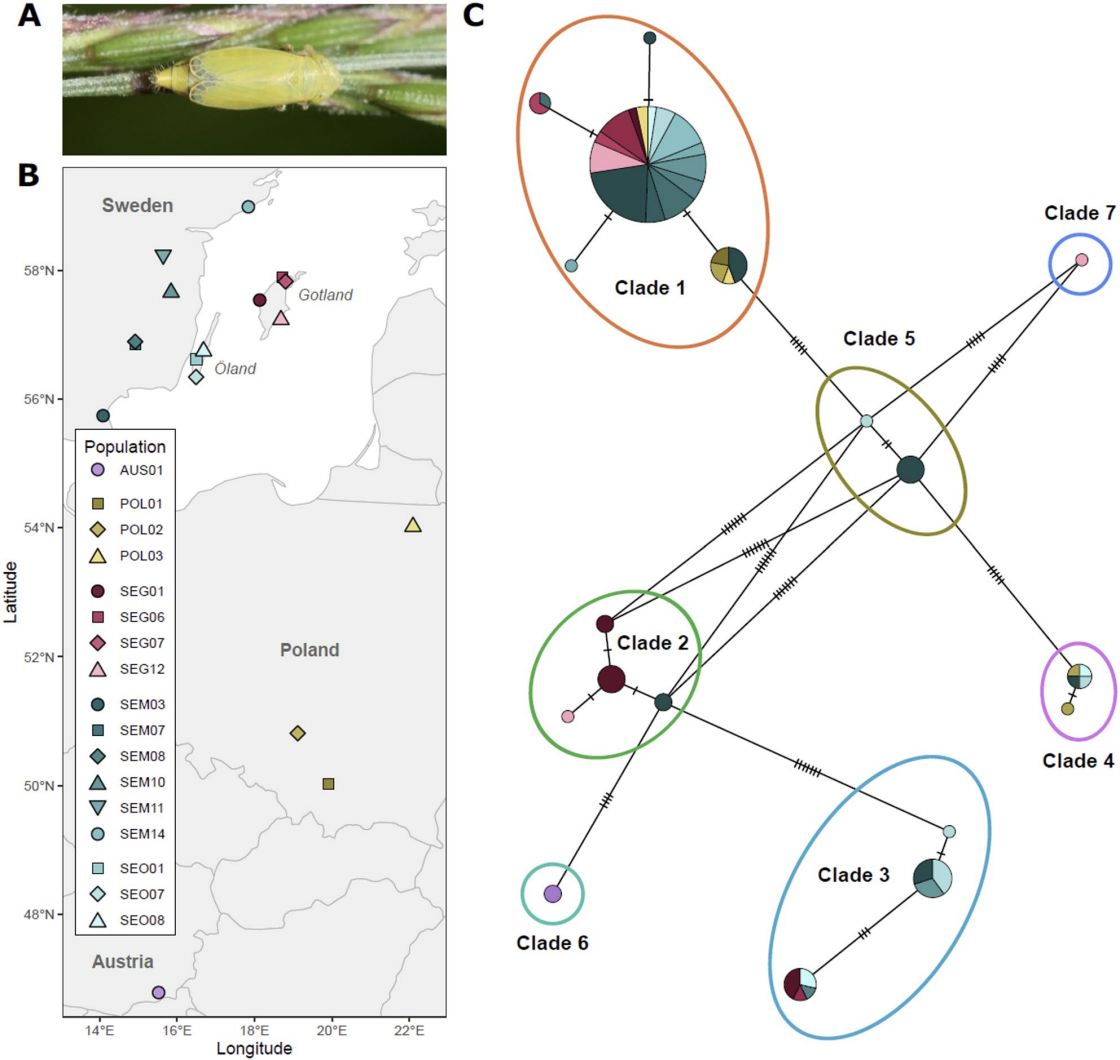
Geographic distribution and genetic diversity of *Verdanus* populations. **A** Specimen of *Verdanus* sp. photographed by Emanuel Kern, via iNaturalist, licensed under CC BY 4.0. **B** Map of the sampled leafhopper populations. **C** Minimum spanning haplotype network of the sampled *Verdanus* diversity. Individual pie charts represent leafhopper COI genotypes, coloured by collection site – as in panel B

Adult specimens of *Verdanus* leafhoppers were collected in 2015 and 2022 from multiple meadow and grassland sites across Sweden, Poland, and Austria (Fig. 1A, B, Table S1, S2). Sampling covered different habitats and populations from both the mainland and the Baltic islands to maximize the likelihood of capturing geographic and ecological variation. Specimens were captured using sweep nets, immediately transferred to 96% ethanol, and stored at -20 °C until processing. Individuals were morphologically identified as *V. abdominalis* following standard diagnostic keys [30, 32]. Voucher specimens were deposited at the Institute of Zoology and Biomedical Research, Jagiellonian University.

### Amplicon-based insect diversity and microbiota profiling

DNA extraction and library preparation were performed at the Institute of Environmental Sciences and the Institute of Zoology and Biomedical Research, Jagiellonian University, following the detailed protocols available online [33].

Briefly, individual insects were placed in 2 mL screw-cap tubes containing lysis buffer (0.4 M NaCl, 10 mM Tris-HCl, 2 mM EDTA, 2% SDS) with proteinase K and ceramic beads. Samples were homogenized and then incubated at 55 °C for 2 h. One-fifth of each homogenate was spiked with 20,000 copies of the artificial 16S rRNA plasmid Ec5502 [34], then purified using SPRI magnetic beads. Amplicon libraries were prepared in a two-step PCR workflow. In the first step (PCR1), a mixture of primers targeting multiple regions – with Illumina adapter overhangs – was used to simultaneously amplify six marker regions: the insect COI barcode region, bacterial 16S rRNA V4 and V1-V2 regions, and fungal ITS1, ITS2, and 18S rRNA V7-V8 region (Table S3). The project was run in two batches: in the first batch, we only used COI, 16S V4, ITS2, and 18S rRNA primers, and in the second batch, we additionally targeted ITS1 and 16S V1-V2; hence, only the first four markers are available for some samples. PCR1 products were checked on agarose gels and purified with SPRI beads, and then used as template for the second PCR, when sample-specific Illumina index combinations were added. The resulting libraries were verified again by gel electrophoresis and pooled for sequencing on an Illumina NextSeq 2000 using 600-cycle P2 flow cells at the Małopolska Centre of Biotechnology of Jagiellonian University. To monitor contamination, negative controls were included at DNA extraction, PCR, and indexing steps (two per type per plate), alongside positive PCR controls.

### Amplicon data analysis

Amplicon sequencing data were processed following the workflow described previously (https://github.com/KarolHub/Phorid-Microbiota*)* [35]. Demultiplexed reads were split into separate datasets based on primer sequences that were then trimmed. Next, sequences were processed using a custom script incorporating PEAR [36], VSEARCH [37], UNOISE3 [38], and USEARCH [39]. Reads were quality filtered, forward and reverse reads were merged, and the resulting contigs were dereplicated and denoised. The resulting zero-radius taxonomic units (zOTUs) (practical equivalent of amplicon sequence variants (ASVs)) were then grouped into OTUs using a 97% similarity threshold. Taxonomic assignment was performed using the SINTAX algorithm against target-appropriate databases: MIDORI v. 239 [40] for host COI data, SILVA v.138 [41, 42] for bacterial 16S rRNA, and UNITE v. 6.0 for fungal ITS1 and ITS2.

COI amplicon data were filtered based on barcode zOTU read abundance (minimum 20 reads), expected sequence length (418 bp), chimera status, the presence of frameshifts or stop codons, and taxonomic assignment, with negative controls and non-target sequences removed. Parasitoid-derived COI sequences were identified during taxonomic filtering but excluded prior to leafhopper genotype inference to ensure that host barcodes reflected the mitochondrial genotype of the insect host rather than any potential parasitoids (Dryinidae, Mymaridae, Pipunculidae, Tachinidae, or Strepsiptera) [43].

Haplotype networks were constructed using PopART v1.7 (https://popart.maths.otago.ac.nz/; [44]) based on host COI barcode sequence alignments and population-based trait matrices. Haplotypes differing by at least four substitutions were assigned to separate clades.

16S rRNA V4 datasets were further decontaminated using a custom script that incorporates information from negative controls included at each stage of laboratory processing, removing potential reagent-derived contaminants based on their relative abundance in samples, as well as non-bacterial reads and sequences corresponding to experimental spike-ins. For visualization purposes, zOTUs detected in fewer than two samples or showing very low cumulative abundance across samples (sum of per-sample relative abundances < 0.01) were collapsed into an “Other” category.

### Metagenomic sequencing, assembly, and annotation

To characterize their dominant symbionts, we sequenced metagenomes from seven *Verdanus* specimens representing four dominant clades, collected from mainland Sweden (4), the Swedish islands of Gotland (1) and Öland (1), and from Austria (1) (Table S2). The specimens were chosen to encompass variation in microbiome composition between geographically separated populations representing various clades, as inferred from the amplicon data. Metagenomic libraries were prepared from the same DNA extracts as amplicon libraries, using the NEBNext UltraExpress FS DNA Library Prep Kit (New England Biolabs, USA) and following the manufacturer’s protocol, but with reagent volumes reduced by half. Sequencing was conducted by Novogene Ltd. on an Illumina NovaSeq X platform, generating paired-end reads (2 × 150 bp), and at Małopolska Centre of Biotechnology of Jagiellonian University using Illumina NextSeq 2000 platform (2 × 300 bp).

Metagenomic data were initially analyzed using PhyloFlash v3.4 [45], which reconstructs small subunit (SSU) rRNA genes directly from metagenomes and assesses their identity based on the SILVA rRNA reference database. Following quality and adapter trimming with Trim Galore v0.6.4 (https://github.com/FelixKrueger/TrimGalore), contigs were then assembled using SPAdes v4.2 (maximum k-mer size = 281) [46] or MEGAHIT v1.2.9 (maximum k-mer size = 255), depending on which method provided complete circular genomes of ancient symbionts [47]. Assembled contigs were taxonomically identified using BLASTn searches against the NCBI nt database, and contigs were compared among the alternative assemblies to resolve the organization of the genomes, especially within the low-GC-contents region of *Nasuia* genome with lower read coverage, and ensure the circular mapping of contigs. This strategy enabled the reconstruction of complete circular *Sulcia* and *Nasuia* genomes, which were rearranged to the same orientation and start position (*Sulcia* - gene lipB; *Nasuia* - hisG) for all analyzed *Verdanus* specimens.

The completeness of contigs corresponding to the additional symbionts *Symbiopectobacterium*, *Rickettsia*, and *Tisiphia* in the selected metagenomes was assessed using CheckM2 [48]. The annotation of *Symbiopectobacterium*, *Rickettsia*, and *Tisiphia* contigs was performed using Prokka [49]. *Sulcia* and *Nasuia* genomes were annotated using a custom workflow,

*(*https://github.com/AnnaMichalik22/Planthopper-ancient-symbioses---supplementary-materials*),* originally developed for the annotation of cicada symbionts [6] and recently adopted for the annotation of the ancient symbionts of other Auchenorrhyncha [8]. The script identifies all Open Reading Frames (ORFs) and translates them into amino acid sequences, which are then iteratively searched using HMMER v3.3.1 against a custom database containing manually curated sets of protein-coding, rRNA, and noncoding RNA (ncRNA) gene alignments from previously characterized *Sulcia* or *Nasuia* lineages. The reference datasets were validated and updated after each annotation round. Detection of rRNA and ncRNA genes was carried out using nhmmer (HMMER v3.3.1) [50], while tRNA genes were identified using tRNAscan-SE v2.0.7 [51].

Reconstructed *Sulcia* and *Nasuia* genomes were visualized using Proksee [52]. To compare genome organization and content between *Verdanus* symbionts and previously published references, we selected *Sulcia* and *Nasuia* genomes that were uploaded to the NCBI database as complete and contained a low proportion of unresolved nucleotide positions, representing a range of host insect species. Comparative synteny plots were generated and illustrated using Promer v3.0.7 and Mummerplot v3.5 [53], and functional analysis visualizations were produced using Processing v. 3.5.4. The resulting figures were subsequently edited in Inkscape v. 1.4.2.

### Microscopy and histological validation

Microscopy was performed to examine the organization and localization of symbiotic microorganisms within *Verdanus abdominalis* host tissues. Females from a Swedish (Öland) population SEO03 were processed at the Institute of Zoology and Biomedical Research, Jagiellonian University, following the general protocols detailed in [54].

For histology and transmission electron microscopy (TEM), dissected abdomens of adult females were fixed in 2.5% glutaraldehyde in 0.1 M phosphate buffer (pH 7.4) and stored for one month. Subsequently, they were rinsed in the same buffer with the addition of sucrose (5.8 g/100 ml) and postfixed in 1% osmium tetroxide for 2 h at room temperature. Fixed tissues were washed in tap water, dehydrated through a graded ethanol series, and embedded in Epon 812 epoxy resin. Semi-thin Sect.  (1 μm) were stained with 1% methylene blue in 1% borax and examined under a Nikon Eclipse 80i light microscope equipped with a digital camera. For ultrastructural studies, ultrathin sections (~ 90 nm) were cut with a Leica EM UC7 ultramicrotome, mounted on copper grids, stained with uranyl acetate and lead citrate, and observed under a Jeol JEM 2100 transmission electron microscope operating at 80 kV.

In addition, for some specimens, fluorescence in situ hybridization (FISH) was performed to confirm the localization of bacterial symbionts. Bacteriome and reproductive tissues dissected from ethanol-preserved specimens were embedded in Technovit 8100 resin and then cut into semithin Sect.  (1 μm thick). Sections were hybridized overnight at room temperature with *Sulcia*-specific (Sul664-Cy5) and *Nasuia*-specific (Bet940-Cy3) oligonucleotide probes [55, 56] with a final concentration of 100 nM in a hybridization buffer containing 1 M Tris-HCl (pH 8.0), 5 M NaCl, 20% SDS, formamide, and distilled water. After hybridization, the slides were washed in PBS three times, dried, and covered with ProLong Gold Antifade Reagent (Life Technologies). Fluorescence signals were visualized using a Zeiss LSM 710 confocal laser scanning microscope.

## Results

### Amplicon sequencing resolves insect genetic structure and highlights the dominance of ancient symbionts

**Fig. 2:**
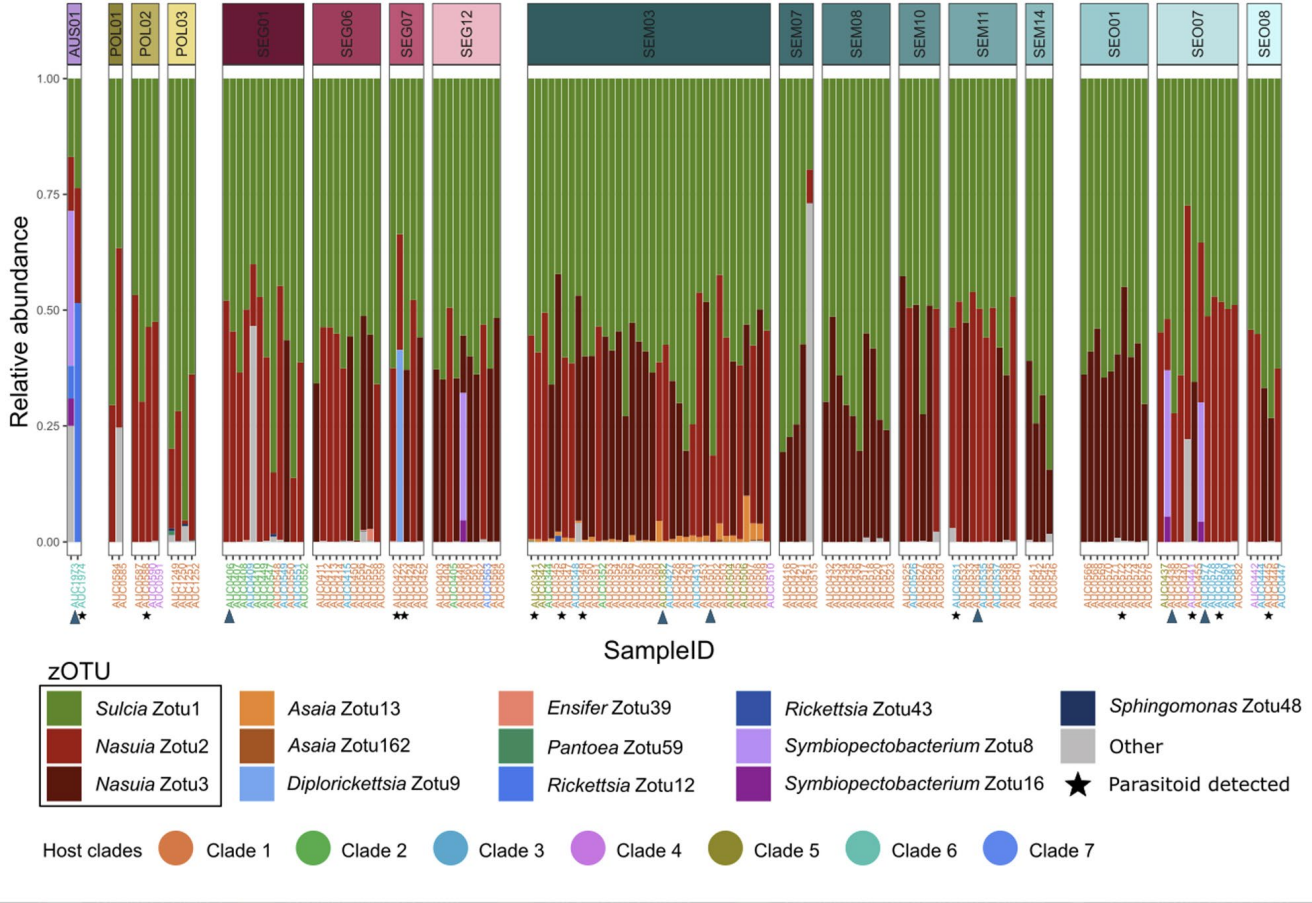
The relative abundance of dominant bacterial zOTUs across individual leafhoppers from 17 populations. Sample labels are coloured according to the clades shown in the haplotype network in Fig. 1C. Triangles indicate specimens used for metagenomics

For the 147 insects successfully processed using marker gene amplicon sequencing, we obtained an average of 5,044 reads for the insect mitochondrial marker COI, 53,141 reads for the conserved bacterial 16S rRNA V4 region, 16,240 reads on average for the 16S rRNA V1-V2 region, and a combined average of 5,720 reads across the three fungal markers (ITS1, ITS2 and 18S rRNA) (Table S2). COI amplicon data allowed us to resolve specimen identity and within-species genetic structure and identify cases of parasitoid infections (Fig. 1B, Tables S4, S5). Bacterial 16S rRNA V4 region data, largely supported by V1-V2 region data, informed us about the dominance of ancient bacterial symbionts *Sulcia* and *Nasuia* and sporadic distributions of other bacteria (Fig. 2, Tables S6-S7). Fungal amplicon data (Tables S8-S10) were dominated by fungi representing the genera *Davidella* (ITS1) and *Cladosporium* (ITS2, 18S rRNA), which were present in all samples. Other fungal taxa were generally of lower abundance and more variable, showing patterns likely related to sampled habitats (Fig. S2-S4).

COI amplicon sequencing identified 240 *Verdanus* zOTUs. Dominant zOTUs in individual libraries generally accounted for ≥ 60% of reads and were used as host barcodes (18 genotypes; Table S2). These barcode sequences differed from each other by up to 17 nucleotides across the 418 bp fragment (~ 4.06% divergence) (Table S5). Haplotype network analysis revealed seven clades separated by at least four nucleotide substitutions (Fig. 1C). The largest number of samples (105) was assigned to clade 1, with other clades represented by 1–18 insects. The distribution of COI haplotypes or clades was not shaped by geography, as the same haplotypes occurred across different populations and regions, and at the same time, individuals sampled at the same location frequently represented different clades. For example, the most common haplotype within clade 1 was represented by at least one individual in all 16 examined Polish or Swedish populations, while within the most comprehensively sampled population, SEM03, we identified seven genotypes representing five clades. In turn, specimens originating from the Austrian population formed a distinct lineage (clade 7).

Twelve *V. abdominalis* individuals were found to be infected with parasitoids from the families Dryinidae (genus *Gonatopus*, 2 individuals) and Pipunculidae (genera *Clistoabdominalis*, *Pipunculus*, and *Tomosvaryella*, 10 individuals). Across infected individuals, parasitoid-derived reads accounted for an average of 43.8% of the COI library (Table S4).

The 16S rRNA – V4 amplicon sequencing data comprised 287 zOTUs above our threshold abundance, which clustered into 178 OTUs (Table S5). Of these, the single detected *Sulcia* zOTU comprised 58.4% of data in a library on average. The rapidly evolving co-symbiont *Nasuia*, represented by two zOTUs in our dataset (one zOTU per individual, with the less common zOTU3 seen in only some of Clade1 individuals), comprised 38.1% of data in a library on average. These two ancient obligate symbionts were present, and typically highly dominant, in all examined individuals (Fig. 2).

Other bacterial zOTUs that met the visualization criteria (detected in at least two samples, and with average relative abundance exceeding 1/15000th) included *Asaia*, *Diplorickettsia*, *Ensifer*, *Pantoea*, *Rickettsia*, *Symbiopectobacterium*, and *Sphingomonas*. Although some of these zOTUs reached higher relative abundances in individual hosts, they were generally sporadic and together accounted for only 1.99% of reads in a library on average. For example, *Symbiopectobacterium* was abundant in four individuals from three populations, with its two zOTUs always co-occurring within the same insects, likely reflecting the presence of different copies of the 16S rRNA gene within the genome of a single bacterium. In turn, the bacterium *Asaia* showed clear geographic patterns, being present in all individuals from the SEM03 population, and rarely elsewhere. *Rickettsia* was represented by two genotypes, one of which was present only in individuals from the Austrian population. Only one specimen in our collection (AUC0585) harbored a plant-pathogenic bacterium *Phytoplasma*. Its high relative abundance in this specimen suggests that *Verdanus* may vector plant pathogens (Fig. S1). Known insect symbionts in the genera *Wolbachia* and *Arsenophonus* were found only in single specimens and at very low abundance (Fig. S1). The microbiota of parasitoid-infected individuals were broadly comparable to those of uninfected hosts. However, we noted that seven of fourteen leafhopper specimens harboring *Rickettsiales* (i.e., *Rickettsia*, *Diplorickettsia*, *Tisiphia*, or *Wolbachia*) were parasitized, suggesting parasitoids as the actual hosts of some of these bacteria.

### Metagenomics resolves *Verdanus* symbiotic system composition

**Fig. 3:**
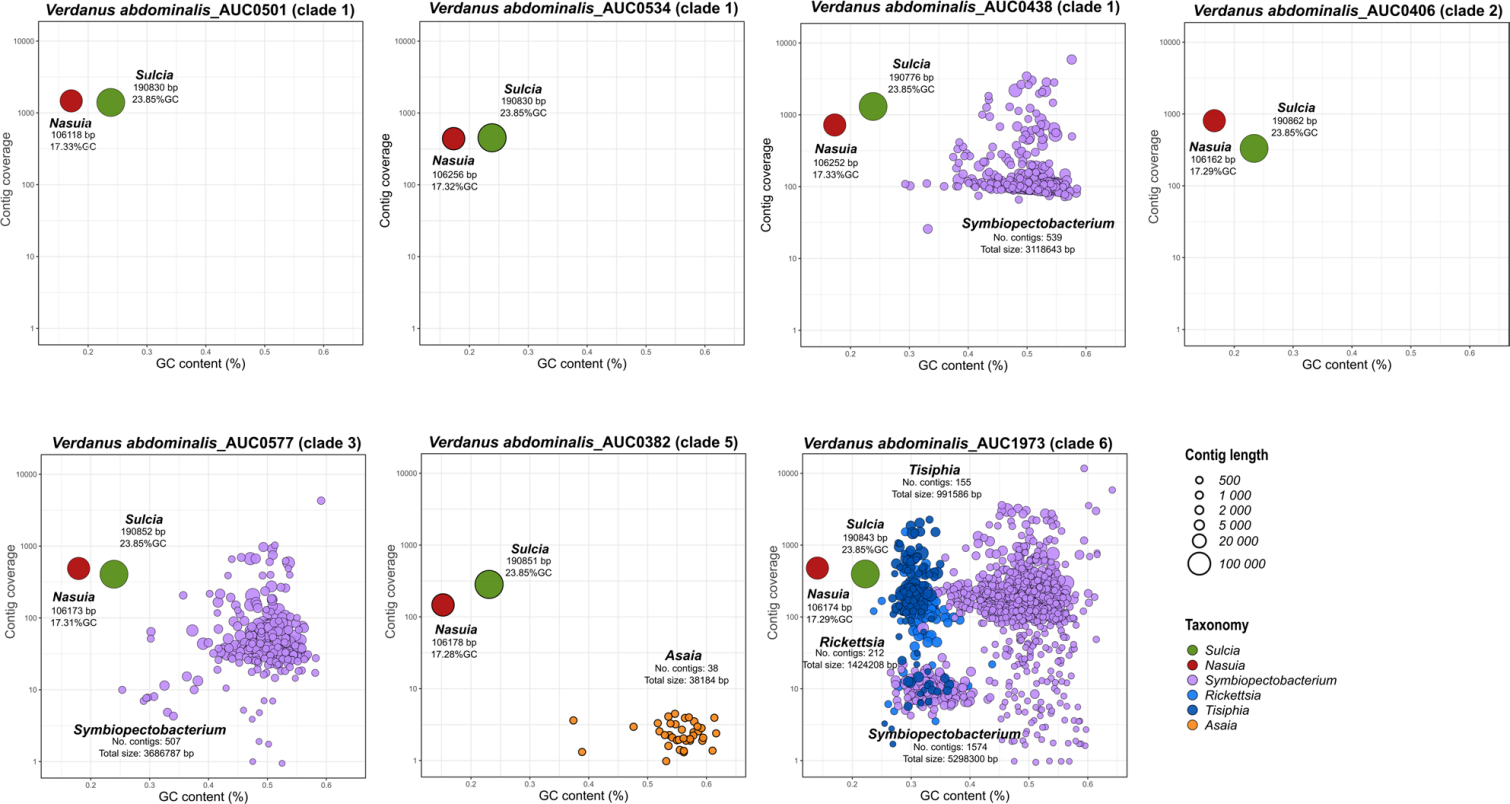
The visualization of symbiont contigs from *Verdanus* specimens representing different COI clades in GC contents and coverage space, with circle color reflecting the assigned taxonomy, and circle size - contig size

PhyloFlash-based reconstruction of ribosomal rRNA sequences from metagenomic datasets, as well as the comparative analyses of metagenomic assemblies, confirmed that the microbial communities of all seven specimens sequenced were dominated by the obligate endosymbionts *Sulcia* and *Nasuia* (Fig. 3). Additionally, in agreement with amplicon data, assemblies for specimens AUC0438, AUC0577, and AUC1973 contained high-coverage *Symbiopectobacterium*, while specimen AUC1973 also contained *Rickettsia* and *Tisiphia* (Fig. 3). In turn, while two of the processed insects contained *Asaia* according to amplicon data, from only one of them, AUC0382, we recovered a low-coverage incomplete genome of this bacterium.

**Fig. 4:**
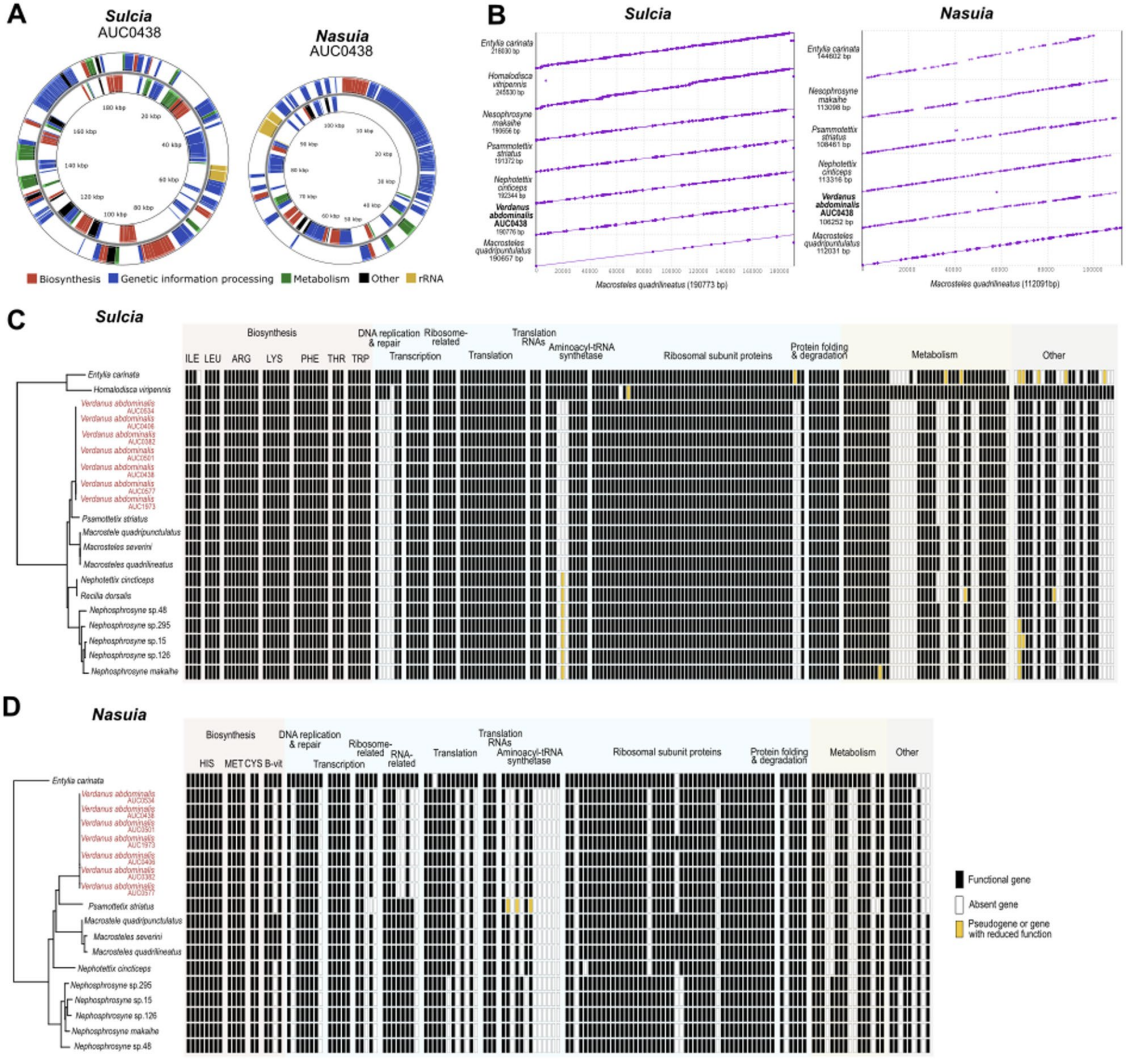
**A** The visualization of complete genomes of representative *Sulcia* and *Nasuia* strains from *Verdanus abdominalis* specimen AUC0438. **B** Protein alignments among *Sulcia* and *Nasuia* genomes from *Macrosteles quadrilineatus* (set as a reference), *Verdanus abdominalis*, and selected other Membracoidea. **C**, **D** The list of non-hypothetical protein-coding and RNA (other than tRNA) genes identified in the genomes of *Sulcia* and *Nasuia* from *Verdanus abdominalis* and selected other Membracoidea. The classification (apparently functional, putatively pseudogenized, or absent) of each gene is based on the length of the ORF relative to that in the genomes from *M. quadrilineatus*

From all seven *Verdanus* metagenomes, we recovered complete circular genomes of both ancient symbionts, showing consistent genome sizes and base composition (Figs. 3 and 4A). *Sulcia* genome sizes ranged from 190,776 to 190,862 bp, with GC contents of 23.85% in all cases. *Nasuia* genome sizes were 106,118 − 106,256 bp, with GC contents of 17.28–17.33%. (Figures 3 and 4A, Table S11). Both *Sulcia* and *Nasuia* genomes were perfectly syntenic with those from other *Verdanus* specimens or previously characterized leafhoppers and treehoppers, with no rearrangements in genome organization detected (Fig. 4B).

In turn, the genomes of *Symbiopectobacterium* identified in the samples AUC0438, AUC0577, and AUC1973 were highly fragmented, represented by 539, 507 and 1574 contigs with a total size 3,118,643; 3,686,787 and 5,298,300 bp, respectively (Fig. 3). In specimen AUC1973, the *Symbiopectobacterium* contigs appear to represent two distinct strains, as indicated by differences in GC content and coverage among contigs. Analysis using CheckM2 tool indicated that these *Symbiopectobacterium* genomes were largely complete (completeness 97.2–100%, contamination ≤ 0.7%) (Table S11). *Rickettsia* and *Tisiphia* assemblies from the specimen AUC1973, with sizes 1,424,208 and 991,586 bp, were less complete (87.73% and 42.89%, respectively). The fragmentary genome of *Asaia* (38 kb, 3.5% completeness) was not considered further.

### Comparative genomics of *Sulcia* and *Nasuia* genomes

Comparative analysis of the newly reconstructed *Sulcia* genomes and previously published references from other Membracoidea (Table S11) revealed a conserved gene content (Fig. 4C, Table S12). All newly reconstructed genomes encoded 193 protein-coding genes, a number consistent with previously studied Deltocephalinae *Sulcia* strains that typically harbor between 191 and 195 protein-coding genes. Functional annotation and comparison among leafhopper-*Sulcia* strains indicated that their gene repertoire is strongly biased toward core biosynthetic processes and genetic information-processing. Specifically, *Sulcia* strains almost universally retained the set of 43 genes comprising complete pathways for the biosynthesis of eight of the ten essential amino acids required by the host: threonine, valine, leucine, isoleucine, lysine, phenylalanine, tryptophan, and arginine (Fig. 4C, Table S12).

Likewise, the genetic information processing machinery was well conserved among the Deltocephalinae strains included in comparisons, but reduced relative to other Membracoidea strains. Specifically, Deltocephalinae-*Sulcia* retained only three genes associated with DNA replication and repair - *dnaE*, *mutS*, and *mutL*, compared to seven in related lineages. They also all retained eight aminoacyl-tRNA synthetase genes, as compared to eleven in other strains. Genes involved in transcription and translation were conserved across samples, highlighting the retention of essential information-processing functions in these highly reduced genomes. For example, of the total of 78 ribosomal protein genes or other genes involved in ribosome assembly and in translation, all but four were universally retained (Fig. 4C, Table S12).

Further, all *Sulcia* strains included in comparisons retained genes essential for primary metabolism and energy production, including the complete set of ATP synthase genes (*atpABCDEFGH*) as well as key genes of the tricarboxylic acid (TCA) cycle (*sucAB*,* aceEF*, and *lpdA*). All of them also contained the *suf* operon (*sufBCDES*), which is involved in iron–sulfur (Fe–S) cluster assembly. Notably, only four genes across the entire *Sulcia* genome were annotated as hypothetical proteins of unknown function, indicating that the vast majority of retained genes correspond to well-characterized and essential cellular processes (Table S12).

The genomes of *Nasuia* are more reduced than those of *Sulcia*, with this reduction evident in both genome sizes and the numbers of protein-coding genes (Fig. 4D, Table S13). *Verdanus*-associated *Nasuia* ranks among the smallest *Nasuia* genomes described to date, both in terms of total genome size and gene content. *Verdanus*-associated *Nasuia* encodes 125 or 126 protein-coding genes, near the lower end of the spectrum for the published genomes (range: 123–144 protein-coding genes). All Deltocephalinae-*Nasuia* genomes are substantially functionally reduced relative to the membracid genome included in the comparisons, with losses spanning all functional categories except for amino acid biosynthesis pathways (methionine and histidine) and transcription-related genes. Further, strains from different Deltocephalinae clades varied substantially in which genes they retained. Compared to other clades, *Verdanus* symbionts have uniquely lost four of otherwise conserved RNA-related genes (*gatA*, *gatB*, *miaB*, *rsmA*), and a greater share of aminoacyl-tRNA synthetases, retaining barely four genes in that category. In turn, gene losses in B vitamin biosynthetic pathways, among ribosomal proteins, or in metabolic functions, are generally shared with at least some other Deltocephalinae-associated strain (Fig. 4D, Table S13).

*Symbiopectobacterium* strains accompanying the ancient symbionts *Sulcia* and *Nasuia* in specimens AUC0438, AUC0577, and AUC1973 possess genomes reduced relative to several of the strains studied before [57, 58], although relying on short-read data likely led to collapse of repetitive contents and omission of some genes [58]. These *Symbiopectobacterium* strains retain nearly complete pathways for the biosynthesis of all ten essential amino acids and most B vitamins (Table S14).

### Tissue distribution and ultrastructural characteristics of ancestral *Verdanus* symbionts

**Fig. 5:**
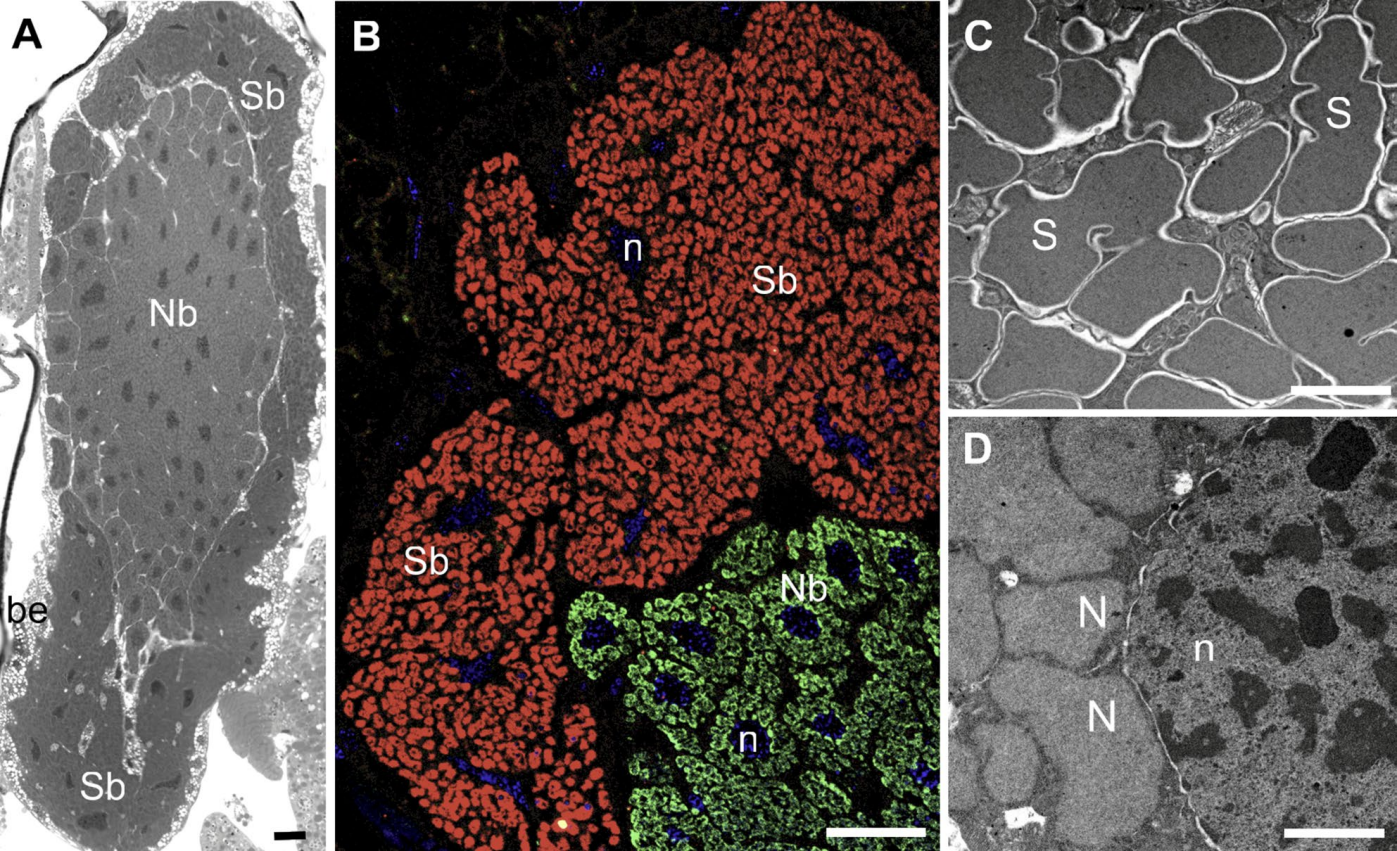
The localization of symbionts in *Verdanus abdominalis* bacteriome. **A** Overall organization of the bacteriome, showing a central region composed of bacteriocytes harboring *Nasuia* (Nb), surrounded by an outer layer of bacteriocytes containing *Sulcia* (Sb). **B** Fluorescence in situ hybridization (FISH) localizes *Sulcia* (red) and *Nasuia* (green) within their respective bacteriocytes within the bacteriome. **C** The ultrastructure of *Sulcia* cells (S) within bacteriocytes. **D** The ultrastructure of *Nasuia* cells (N) within bacteriocytes. n - nucleus; scale bars in A, B − 20 μm, C, D − 2 μm

Microscopic analyses demonstrated a conserved localization and spatial organization of the obligate bacterial symbionts *Sulcia* and *Nasuia* in *Verdanus abdominalis* specimens, closely resembling that in previously studied leafhoppers (Fig. 5). In all examined individuals, both symbionts were consistently housed within two paired bacteriomes situated in the abdominal cavity, adjacent to the body wall (Fig. 5A, B). Each bacteriome exhibited a well-defined internal organization, comprising two well-distinguishable zones. The inner region was occupied by bacteriocytes containing *Nasuia*, which were surrounded by a peripheral zone composed of bacteriocytes harboring *Sulcia* (Fig. 5A, B). This arrangement of symbiont-containing cells was consistent across all analyzed specimens.

Bacteriocytes hosting either *Sulcia* or *Nasuia* were uninucleate, highly polyploid, and densely packed with symbiont cells (Fig. 5A, B, C, D). Host cell organelles were found in the spaces between symbionts, with mitochondria being the most numerous component. Bacteriocytes associated with *Nasuia* were noticeably smaller than those containing *Sulcia* (Fig. 3A, B). Cells of both symbionts were surrounded by three membranes: two of bacterial origin and an outer perisymbiotic membrane derived from the host. Moreover, *Sulcia* cells exhibited greater electron density than *Nasuia* cells (Fig. 5C, D).

## Discussion

### Cryptic host diversity contrasted with symbiotic stability

COI amplicon sequencing revealed extensive cryptic mitochondrial diversity within morphologically identified *Verdanus abdominalis*, with multiple divergent lineages co-occurring locally and showing little geographic structuring. This pattern contrasts with some previously studied Auchenorrhyncha systems, where mitochondrial variation is often geographically structured, as in *Philaenus* spittlebugs [59], or dominated by a single mitochondrial variant, as reported for *Macrosteles laevis* [29]. Together with the absence of clear correspondence between haplotypes and sampling localities, these data suggest that *V. abdominalis* should be considered a species complex rather than a single panmictic taxon. Indeed, the level of variation encountered (4.06% among the most dissimilar zOTUs) exceeds the canonical 3% threshold for species delimitation [60], shown to perform well for most Hemiptera [61]. However, in some cases, within-species diversity may exceed that threshold [62]; hence, other types of data are needed to resolve the biological nature of the observed variation. Systematic data linking morphology and genetic diversity are currently lacking for the *Verdanus/Diplocolenus* clade [60]. Our results, therefore, highlight the need for integrative taxonomic approaches combining morphology with mitochondrial and nuclear markers, as well as broader biogeographic sampling, to resolve species boundaries and evolutionary relationships in this group [63, 64] - similarly to other dark taxa [35, 65] .

Despite pronounced host-level genetic heterogeneity, all examined individuals consistently harbored the same two ancient obligate bacterial symbionts, *Candidatus* Sulcia muelleri and *Candidatus* Nasuia deltocephalinicola. These symbionts colonized the ancestors of Membracoidea and are retained in most extant Cicadellidae, including Deltocephalinae to which *Verdanus* belongs [12, 15–17]. Our data demonstrate that host lineage divergence within the *V. abdominalis* species complex has occurred without disruption of this ancestral nutritional symbiosis. The fact that only a single 16S rRNA genotype of *Sulcia* was discovered in our dataset, combined with the high nucleotide identity among gene sequences (Table S15), attests to its impressive evolutionary stability, reported previously from other Auchenorrhyncha systems [6, 8]. *Nasuia* evolves much more quickly, as reflected by the greater nucleotide sequence dissimilarity among the genomes of *Verdanus*-derived strains (Table S16) and the presence of distinct *Nasuia* S rRNA variants in the amplicon data for some individuals. The reasons for such discrepancy in evolutionary rates, and how such variation emerges, are unclear [8, 64, 66]. Nevertheless, in our system, it did not translate to functional divergence.

In contrast to the universal presence of *Sulcia* and *Nasuia*, additional bacterial associates were rare, unevenly distributed among individuals, or population-specific, consistent with facultative, opportunistic, or transient associations. The scattered occurrence of *Symbiopectobacterium* and the geographically structured distributions of *Asaia* stand in sharp contrast to the ubiquitous obligate symbionts, or else, of highly diverse and dynamic facultative microbiota of hemipterans such as aphids [67, 68]. The low prevalence and scattered distribution of these secondary microbes, combined with ancient symbionts’ gene contents conservation, suggest they are unlikely to provide critical functions. This indicates that the essential nutritional roles in *Verdanus* are primarily fulfilled by *Sulcia* and *Nasuia*, unlike in some other hemipterans (e.g., planthoppers), where additional symbionts contribute more substantially [8].

On the other hand, non-essential symbionts can provide a wide range of benefits that could improve host insects’ fitness under particular conditions [10], including defense against diverse natural enemies and abiotic stressors, or the ability to feed on particular host plants. These effects, well-researched in model systems such as aphids [67, 68] but poorly understood in Auchenorrhyncha, could lead to rapid spread and perhaps local fixation of beneficial symbionts, resembling the patterns observed in the *Asaia*-carrying SEM03 population. However, reproductive parasitism could have similar effects [69]. On the other hand, the sporadic detection of *Symbiopectobacterium* may reflect an early stage of host population invasion by potentially heritable symbiont providing nutritional or other benefits [58, 70]. However, given the broader environmental distribution of this clade, it could also indicate transient or environmentally acquired associations, possibly linked to plant-associated bacteria or pathogenicity [70]. Similarly, *Rickettsia* could play a wide range of roles in the leafhopper host biology [71–73] - although in our system, it likely associates with parasitoids (see Sect.  4.3).

### Long-term stability of the Sulcia-Nasuia dual symbiosis

Amplicon sequencing demonstrated the universal co-occurrence and dominance of *Sulcia* and *Nasuia* across all populations and host clades that we examined. Genomic analyses further showed that symbiont genomes recovered from different *Verdanus* individuals were highly similar to one another. Also, comparative analyses against previously published leafhopper symbiont genomes revealed perfect synteny and strong conservation of gene content in both bacteria.

In terms of size, the *Sulcia* genomes from *Verdanus* (~ 191 kb) fall within the range reported for other leafhopper-associated strains, whereas the *Nasuia* genomes (~ 106 kb) are the smallest described to date [12, 16, 17]. Despite the extreme genome reduction of the latter, functional annotation confirmed retention of the canonical, complementary essential amino acid biosynthesis pathways, with *Sulcia* encoding eight and *Nasuia* contributing the remaining two [12]. The conservation of these pathways across divergent hosts indicates strong purifying selection acting on symbiont metabolic functions, consistent with irreversible host dependence and tight functional integration [74, 75]. Independent anatomical evidence further reinforces this conclusion. Light, fluorescence, and electron microscopy revealed a bacteriome organization in *Verdanus* that closely resembles that described for other deltocephaline leafhoppers, such as *Macrosteles quadrilineatus* [12, 15]. This conserved spatial arrangement mirrors their functional division of labor and suggests long-term co-adaptation at the cellular and tissue levels.

### Robustness of the symbiosis to biotic interactions

Biological interactions such as parasitism can influence host microbiota and complicate their interpretation. For example, in *Philaenus* spittlebugs, the presence of parasitoids has correlated with that of a specific *Wolbachia* lineage, implicating parasitoids as true hosts of that endosymbiont [59]. In our study, multitarget amplicon sequencing similarly enabled the detection of parasitoid DNA in a subset of *Verdanus* individuals, demonstrating the sensitivity of this approach for uncovering hidden ecological interactions. The higher prevalence of Rickettsiales bacteria among parasitoid-positive *Verdanus* individuals suggested parasitoids as the source of at least some of these strains, but the low parasitoid and Rickettsiales prevalence and their substantial diversity hampers the statistical assessment of these interactions. While parasitoids may contribute their own symbionts to the overall microbial community profile, it does not appear that such infections consistently alter leafhoppers’ specialized microbiota [29].

## Conclusions and broader evolutionary implications

Taken together, compositional, genomic, and anatomical evidence converge on a model in which ancient nutritional symbioses in deltocephaline leafhoppers, including *Verdanus*, represent exceptionally stable evolutionary systems. These associations persist across host speciation, cryptic diversification, and occasional co-infections by other microbes. The combination of extreme genomic reduction, conserved metabolic function, and conserved tissue organization suggests that evolutionary change in these symbioses is strongly constrained, with limited scope for symbiont turnover or functional replacement. More broadly, our results underscore the importance of integrating host genetics, symbiont genomics, and cellular biology to understand the tempo and mode of evolution in obligate insect-microbe partnerships.

## Supplementary Information

Supplementary Material 1.

Supplementary Material 2.

## Data Availability

Collection metadata and specimen details are provided in Supplementary Tables S1-S2. Specimen occurrence and barcode sequence data have also been uploaded to the Barcode of Life Database (part of the dataset DS-PIEWI). Raw amplicon sequencing data have been uploaded to NCBI BioProject PRJNA1403467, and curated data are provided in Supplementary Tables S4-S9. Metagenomic data (raw reads and genomic assemblies) can be accessed through BioProjects PRJNA1405624-PRJNA1405626, PRJNA1445917, PRJNA1445918, PRJNA1445919, and PRJNA1445920.

## References

[1] McFall-Ngai M, Hadfield MG, Bosch TCG, Carey HV, Domazet-Lošo T, Douglas AE, et al. Animals in a bacterial world, a new imperative for the life sciences. Proc Natl Acad Sci. 2013;110:3229. 10.1073/pnas.1218525110. 23391737 10.1073/pnas.1218525110 PMC3587249

[2] Sudakaran S, Kost C, Kaltenpoth M. Symbiont acquisition and replacement as a source of ecological innovation. Trends Microbiol. 2017;25:375–90. 10.1016/j.tim.2017.02.014. 28336178 10.1016/j.tim.2017.02.014

[3] Moran NA, McCutcheon JP, Nakabachi A. Genomics and evolution of heritable bacterial symbionts. Annu Rev Genet. 2008;42:165–90. 10.1146/annurev.genet.41.110306.130119. 18983256 10.1146/annurev.genet.41.110306.130119

[4] Cornwallis CK, Van ’T, Padje A, Ellers J, Klein M, Jackson R, Kiers ET, et al. Symbioses shape feeding niches and diversification across insects. Nat Ecol Evol. 2023;7:1022–44. 10.1038/s41559-023-02058-0. 37202501 10.1038/s41559-023-02058-0 PMC10333129

[5] Douglas AE. How multi-partner endosymbioses function. Nat Rev Microbiol. 2016;14:731–43. 10.1038/nrmicro.2016.151. 27795568 10.1038/nrmicro.2016.151

[6] Łukasik P, Nazario K, Van Leuven JT, Campbell MA, Meyer M, Michalik A, et al. Multiple origins of interdependent endosymbiotic complexes in a genus of cicadas. Proc Natl Acad Sci. 2018;115:E226. 10.1073/pnas.1712321115. 29279407 10.1073/pnas.1712321115 PMC5777040

[7] Manzano-Marín A, Coeur d’acier A, Clamens A-L, Cruaud C, Barbe V, Jousselin E. Co-obligate symbioses have repeatedly evolved across aphids, but partner identity and nutritional contributions vary across lineages. Peer Community J. 2023;3. 10.24072/pcjournal.278.

[8] Michalik A, Franco DC, Deng J, Prus-Frankowska M, Stroiński A, Łukasik P. Convergent extreme reductive evolution in ancient planthopper symbioses. Nat Commun. 2026;17:2473. 10.1038/s41467-026-69238-x. 41654530 10.1038/s41467-026-69238-x PMC12992758

[9] Lemoine MM, Engl T, Kaltenpoth M. Microbial symbionts expanding or constraining abiotic niche space in insects. Curr Opin Insect Sci. 2020;39:14–20. 10.1016/j.cois.2020.01.003. 32086000 10.1016/j.cois.2020.01.003

[10] Łukasik P, Kolasa MR. With a little help from my friends: the roles of microbial symbionts in insect populations and communities. Philos Trans R Soc B Biol Sci. 2024;379:20230122. 10.1098/rstb.2023.0122. 10.1098/rstb.2023.0122 PMC11070262 38705185

[11] Wu D, Daugherty SC, Van Aken SE, Pai GH, Watkins KL, Khouri H, et al. Metabolic complementarity and genomics of the dual bacterial symbiosis of sharpshooters. PLOS Biol. 2006;4:e188. 10.1371/journal.pbio.0040188. 16729848 10.1371/journal.pbio.0040188 PMC1472245

[12] Bennett GM, Moran NA. Small, smaller, smallest: the origins and evolution of ancient dual symbioses in a phloem-feeding insect. Genome Biol Evol. 2013;5:1675–88. 10.1093/gbe/evt118. 23918810 10.1093/gbe/evt118 PMC3787670

[13] Mao M, Yang X, Poff K, Bennett G. Comparative genomics of the dual-obligate symbionts from the treehopper, *Entylia carinata* (Hemiptera: Membracidae), provide insight into the origins and evolution of an ancient symbiosis. Genome Biol Evol. 2017;9:1803–15. 10.1093/gbe/evx134. 28854637 10.1093/gbe/evx134 PMC5533117

[14] Moran NA, Tran P, Gerardo NM. Symbiosis and insect diversification: an ancient symbiont of sap-feeding insects from the bacterial phylum Bacteroidetes. Appl Environ Microbiol. 2005;71:8802. 10.1128/AEM.71.12.8802-8810.2005. 16332876 10.1128/AEM.71.12.8802-8810.2005 PMC1317441

[15] Kobiałka M, Michalik A, Szwedo J, Szklarzewicz T. Diversity of symbiotic microbiota in Deltocephalinae leafhoppers (Insecta, Hemiptera, Cicadellidae). Arthropod Struct Dev. 2018;47:268–78. 10.1016/j.asd.2018.03.005. 29621609 10.1016/j.asd.2018.03.005

[16] Waneka G, Vasquez YM, Bennett GM, Sloan DB. Mutational pressure drives differential genome conservation in two bacterial endosymbionts of sap-feeding insects. Genome Biol Evol. 2021;13:evaa254. 10.1093/gbe/evaa254. 33275136 10.1093/gbe/evaa254 PMC7952229

[17] Vasquez YM, Bennett GM. A complex interplay of evolutionary forces continues to shape ancient co-occurring symbiont genomes. iScience. 2022;25:104786. 10.1016/j.isci.2022.104786. 35982793 10.1016/j.isci.2022.104786 PMC9379567

[18] Moran NA, Bennett GM. The tiniest tiny genomes. Annu Rev Microbiol. 2014;68:195–215. 10.1146/annurev-micro-091213-112901. 24995872 10.1146/annurev-micro-091213-112901

[19] McCutcheon JP, Garber AI, Spencer N, Warren JM. How do bacterial endosymbionts work with so few genes? PLOS Biol. 2024;22:e3002577. 10.1371/journal.pbio.3002577. 38626194 10.1371/journal.pbio.3002577 PMC11020763

[20] Michalik A, Castillo Franco D, Kobiałka M, Szklarzewicz T, Stroiński A, Łukasik P. Alternative transmission patterns in independently acquired nutritional co-symbionts of Dictyopharidae planthoppers. mBio. 2021;12:e01228–21. 10.1128/mbio.01228-21. 34465022 10.1128/mBio.01228-21 PMC8406288

[21] Michalik A, Franco C, Szklarzewicz D, Stroiński T, Łukasik A. Facultatively intrabacterial localization of a planthopper endosymbiont as an adaptation to its vertical transmission. mSystems. 2024;e00634–24. 10.1128/msystems.00634-24. 10.1128/msystems.00634-24 PMC11264691 38934538

[22] Gossett JM, Porter ML, Vasquez YM, Bennett GM, Chong RA. Genomic comparisons reveal selection pressure and functional variation between nutritional endosymbionts of cave-adapted and epigean Hawaiian planthoppers. Genome Biol Evol. 2023;15:evad031. 10.1093/gbe/evad031. 36864565 10.1093/gbe/evad031 PMC10030309

[23] Kobiałka M, Michalik A, Walczak M, Szklarzewicz T. Dual bacterial-fungal symbiosis in Deltocephalinae leafhoppers (Insecta, Hemiptera, Cicadomorpha: Cicadellidae). Microb Ecol. 2018;75:771–82. 10.1007/s00248-017-1075-y. 28939987 10.1007/s00248-017-1075-y PMC5856902

[24] Matsuura Y, Moriyama M, Łukasik P, Vanderpool D, Tanahashi M, Meng X-Y, et al. Recurrent symbiont recruitment from fungal parasites in cicadas. Proc Natl Acad Sci. 2018;115:E5970. 10.1073/pnas.1803245115. 29891654 10.1073/pnas.1803245115 PMC6042066

[25] Bennett GM, Abbà S, Kube M, Marzachì C. Complete genome sequences of the obligate symbionts *Candidatus* Sulcia muelleri and *Ca.* Nasuia deltocephalinicola from the pestiferous leafhopper *Macrosteles quadripunctulatus* (Hemiptera: Cicadellidae. Genome Announc. 2016;4:e01604–15. 10.1128/genomeA.01604-15. 26798106 10.1128/genomeA.01604-15 PMC4722273

[26] Moriyama M, Nishide Y, Toyoda A, Itoh T, Fukatsu T. Complete genomes of mutualistic bacterial co-symbionts *Candidatus* Sulcia muelleri and *Candidatus* Nasuia deltocephalinicola of the rice green leafhopper *Nephotettix cincticeps*. Microbiol Resour Announc. 2023;12:e00353–23. 10.1128/MRA.00353-23. 37623315 10.1128/MRA.00353-23 PMC10508130

[27] Ishii Y, Matsuura Y, Kakizawa S, Nikoh N, Fukatsu T. Diversity of bacterial endosymbionts associated with *Macrosteles* leafhoppers vectoring phytopathogenic phytoplasmas. Appl Env Microbiol. 2013;79:5013–22. 23770905 10.1128/AEM.01527-13 PMC3754707

[28] Szklarzewicz T, Świerczewski D, Stroiński A, Michalik A. Conservatism and stability of the symbiotic system of the invasive alien treehopper *Stictocephala bisonia* (Hemiptera, Cicadomorpha, Membracidae). Ecol Entomol. 2020;45:876–85. 10.1111/een.12861.

[29] Mulio SÅ, Zwolińska A, Klejdysz T, Prus-Frankowska M, Michalik A, Kolasa M, et al. Limited variation in microbial communities across populations of *Macrosteles* leafhoppers (Hemiptera: Cicadellidae). Environ Microbiol Rep. 2024;16:e13279. 10.1111/1758-2229.13279. 38855918 10.1111/1758-2229.13279 PMC11163331

[30] Ossiannilsson F. Part 3: The families Cicadidae: Deltocephalinae, catalogue, literature and index. The Auchenorrhyncha (Homoptera) of Fennoscandia and Denmark. Scandinavian Science. 1983.

[31] Söderman G, Gillerfors G, Endrestöl A. An annotated catalogue of the Auchenorrhyncha of Northern Europe (Insecta, Hemiptera: Fulgoromorpha et Cicadomorpha). Cicadina. 2009;10:33–69.

[32] Biedermann R, Niedringhaus R. The plant- and leafhoppers of Germany. Identification key to all species. 2009.

[33] Buczek M, Prus-Frankowska M, Łukasik P. Quantitative multi-target amplicon sequencing workflow v1. 2024. 10.17504/protocols.io.36wgq351ylk5/v1

[34] Tourlousse DM, Yoshiike S, Ohashi A, Matsukura S, Noda N, Sekiguchi Y. Synthetic spike-in standards for high-throughput 16S rRNA gene amplicon sequencing. Nucleic Acids Res. 2017;45:e23–23. 10.1093/nar/gkw984. 27980100 10.1093/nar/gkw984 PMC5389483

[35] Nowak KH, Hartop E, Prus-Frankowska M, Buczek M, Kolasa MR, Roslin T, et al. What lurks in the dark? An innovative framework for studying diverse wild insect microbiota. Microbiome. 2025;13:186. 10.1186/s40168-025-02169-9. 40796904 10.1186/s40168-025-02169-9 PMC12341219

[36] Zhang J, Kobert K, Flouri T, Stamatakis A. PEAR: a fast and accurate Illumina Paired-End reAd mergeR. Bioinformatics. 2014;30:614–20. 10.1093/bioinformatics/btt593. 24142950 10.1093/bioinformatics/btt593 PMC3933873

[37] Rognes T, Flouri T, Nichols B, Quince C, Mahé F. VSEARCH: a versatile open source tool for metagenomics. PeerJ. 2016;4:e2584–2584. 10.7717/peerj.2584. 27781170 10.7717/peerj.2584 PMC5075697

[38] Edgar RC. UNOISE2: improved error-correction for Illumina 16S and ITS amplicon sequencing. 2016. 10.1101/081257

[39] Zhou Y, Liu Y-X, Li X. USEARCH 12: Open-source software for sequencing analysis in bioinformatics and microbiome iMeta. 2024;3:e236.10.1002/imt2.236. 10.1002/imt2.236 PMC11487603 39429875

[40] Leray M, Knowlton N, Machida RJ. MIDORI2: A collection of quality controlled, preformatted, and regularly updated reference databases for taxonomic assignment of eukaryotic mitochondrial sequences. Environ DNA. 2022;4:894–907. 10.1002/edn3.303.

[41] Quast C, Pruesse E, Yilmaz P, Gerken J, Schweer T, Yarza P, et al. The SILVA ribosomal RNA gene database project: improved data processing and web-based tools. Nucleic Acids Res. 2013;41. 10.1093/nar/gks1219. Database issue:D590–6. 10.1093/nar/gks1219 PMC3531112 23193283

[42] Yilmaz P, Parfrey LW, Yarza P, Gerken J, Pruesse E, Quast C, et al. The SILVA and All-species Living Tree Project (LTP) taxonomic frameworks. Nucleic Acids Res. 2014;42:D643–8. 10.1093/nar/gkt1209. 24293649 10.1093/nar/gkt1209 PMC3965112

[43] Waloff N, Jeris MA. Communities of parasitoids associated with leafhoppers and planthoppers in Europe. Advances in Ecological Research. Academic; 1987. pp. 281–376. 10.1016/S0065-2504(08)60248-2.

[44] Leigh JW, Bryant D. Popart: full-feature software for haplotype network construction. Methods Ecol Evol. 2015;6:1110–6. 10.1111/2041-210X.12410.

[45] Gruber-Vodicka HR, Seah BKB, Pruesse E, PhyloFlash. Rapid small-subunit rRNA profiling and targeted assembly from metagenomes. mSystems. 2020;5:e00920–20. 10.1128/mSystems.00920-20. 33109753 10.1128/mSystems.00920-20 PMC7593591

[46] Bankevich A, Nurk S, Antipov D, Gurevich AA, Dvorkin M, Kulikov AS, et al. SPAdes: A new genome assembly algorithm and its applications to single-cell sequencing. J Comput Biol. 2012;19:455–77. 10.1089/cmb.2012.0021. 22506599 10.1089/cmb.2012.0021 PMC3342519

[47] Li D, Luo R, Liu C-M, Leung C-M, Ting H-F, Sadakane K, et al. MEGAHIT v1.0: A fast and scalable metagenome assembler driven by advanced methodologies and community practices. Methods. 2016;102:3–11. 10.1016/j.ymeth.2016.02.020. 27012178 10.1016/j.ymeth.2016.02.020

[48] Chklovski A, Parks DH, Woodcroft BJ, Tyson GW. CheckM2: a rapid, scalable and accurate tool for assessing microbial genome quality using machine learning. Nat Methods. 2023;20:1203–12. 10.1038/s41592-023-01940-w. 37500759 10.1038/s41592-023-01940-w

[49] Seemann T. Prokka: rapid prokaryotic genome annotation. Bioinformatics. 2014;30:2068–9. 10.1093/bioinformatics/btu153. 24642063 10.1093/bioinformatics/btu153

[50] Eddy SR. Accelerated profile HMM searches. PLOS Comput Biol. 2011;7:1–16. 10.1371/journal.pcbi.1002195. 10.1371/journal.pcbi.1002195 PMC3197634 22039361

[51] Lowe TM, Eddy SR. tRNAscan-SE: a program for improved detection of transfer RNA genes in genomic sequence. Nucleic Acids Res. 1997;25:955–64. 10.1093/nar/25.5.955. 9023104 10.1093/nar/25.5.955 PMC146525

[52] Grant JR, Enns E, Marinier E, Mandal A, Herman EK, Chen C, et al. Proksee: in-depth characterization and visualization of bacterial genomes. Nucleic Acids Res. 2023;51:W484–92. 10.1093/nar/gkad326. 37140037 10.1093/nar/gkad326 PMC10320063

[53] Kurtz S, Phillippy A, Delcher AL, Smoot M, Shumway M, Antonescu C, et al. Versatile and open software for comparing large genomes. Genome Biol. 2004;5:R12. 10.1186/gb-2004-5-2-r12. 14759262 10.1186/gb-2004-5-2-r12 PMC395750

[54] Michalik A, Castillo Franco D, Deng J, Szklarzewicz T, Stroiński A, Kobiałka M, et al. Variable organization of symbiont-containing tissue across planthoppers hosting different heritable endosymbionts. Front Physiol. 2023;14:1135346. 10.3389/fphys.2023.1135346. 37035661 10.3389/fphys.2023.1135346 PMC10073718

[55] Demanèche S, Sanguin H, Poté J, Navarro E, Bernillon D, Mavingui P, et al. Antibiotic-resistant soil bacteria in transgenic plant fields. Proc Natl Acad Sci. 2008;105:3957–62. 10.1073/pnas.0800072105. 18292221 10.1073/pnas.0800072105 PMC2268783

[56] Koga R, Bennett GM, Cryan JR, Moran NA. Evolutionary replacement of obligate symbionts in an ancient and diverse insect lineage. Environ Microbiol. 2013;15:2073–81. 10.1111/1462-2920.12121. 23574391 10.1111/1462-2920.12121

[57] Yorimoto S, Hattori M, Nozaki T, Shigenobu S. Evolution of multi-partner symbiotic systems in the Cerataphidini tribe: genome reduction of *Buchnera* and frequent turnover of companion symbionts. 2025. 10.1101/2025.07.09.664008

[58] Gunasekaran D, Sicard A, Almeida RPP, Bennett GM. Characterizing a novel *Symbiopectobacterium purcellii* MEX strain at the early stages of establishing a symbiotic relationship. Genome Biol Evol. 2026;evaf252. 10.1093/gbe/evaf252. 10.1093/gbe/evaf252 PMC12813293 41512111

[59] Kolasa M, Kajtoch Ł, Michalik A, Maryańska-Nadachowska A, Łukasik P. Till evolution do us part: The diversity of symbiotic associations across populations of *Philaenus* spittlebugs. Environ Microbiol. 2023;25:2431–46. 10.1111/1462-2920.16473. 37525959 10.1111/1462-2920.16473

[60] Hebert PDN, Cywinska A, Ball SL, deWaard JR. Biological identifications through DNA barcodes. Proc R Soc Lond B Biol Sci. 2003;270:313–21. 10.1098/rspb.2002.2218. 10.1098/rspb.2002.2218 PMC1691236 12614582

[61] Park D-S, Foottit R, Maw E, Hebert PDN. Barcoding bugs: DNA-based identification of the true bugs (Insecta: Hemiptera: Heteroptera). PLoS ONE. 2011;6:e18749. . 10.1371/journal.pone.0018749 21526211 10.1371/journal.pone.0018749 PMC3078146

[62] Virgilio M, Backeljau T, Nevado B, De Meyer M. Comparative performances of DNA barcoding across insect orders. BMC Bioinformatics. 2010;11:206. 10.1186/1471-2105-11-206. 20420717 10.1186/1471-2105-11-206 PMC2885370

[63] Dayrat B. Towards integrative taxonomy. Biol J Linn Soc. 2005;85:407–17. 10.1111/j.1095-8312.2005.00503.x.

[64] Deng J, Bennett GM, Castillo Franco D, Prus-Frankowska M, Michalik A, Łukasik P. Genome comparison reveals inversions and alternative evolutionary history of nutritional endosymbionts in planthoppers (Hemiptera: Fulgoromorpha). Genome Biol Evol. 2023;15. 10.1093/gbe/evad120. 10.1093/gbe/evad120 PMC10333215 37392458

[65] Srivathsan A, Ang Y, Heraty JM, Hwang WS, Jusoh WFA, Kutty SN, et al. Convergence of dominance and neglect in flying insect diversity. Nat Ecol Evol. 2023;7:1012–21. 10.1038/s41559-023-02066-0. 37202502 10.1038/s41559-023-02066-0 PMC10333119

[66] Bennett GM, McCutcheon John P, MacDonald Bradon R, Dwight R, Moran Nancy A. Differential genome evolution between companion symbionts in an insect-bacterial symbiosis. mBio. 2014;5:e01697–14. 10.1128/mBio.01697-14. 25271287 10.1128/mBio.01697-14 PMC4196230

[67] Oliver KM, Degnan PH, Burke GR, Moran NA. Facultative symbionts in aphids and the horizontal transfer of ecologically important traits. Annu Rev Entomol. 2010;55:247–66. 10.1146/annurev-ento-112408-085305. 19728837 10.1146/annurev-ento-112408-085305

[68] Oliver KM, Smith AH, Russell JA. Defensive symbiosis in the real world – advancing ecological studies of heritable, protective bacteria in aphids and beyond. Funct Ecol. 2014;28:341–55. 10.1111/1365-2435.12133.

[69] Kaur R, Shropshire JD, Cross KL, Leigh B, Mansueto AJ, Stewart V, et al. Living in the endosymbiotic world of *Wolbachia*: A centennial review. Cell Host Microbe. 2021;29:879–93. 10.1016/j.chom.2021.03.006. 33945798 10.1016/j.chom.2021.03.006 PMC8192442

[70] Martinson VG, Gawryluk RMR, Gowen BE, Curtis CI, Jaenike J, Perlman SJ. Multiple origins of obligate nematode and insect symbionts by a clade of bacteria closely related to plant pathogens. Proc Natl Acad Sci. 2020;117:31979. 10.1073/pnas.2000860117. 33257562 10.1073/pnas.2000860117 PMC7749356

[71] Liu X-D, Guo H-F. Importance of endosymbionts *Wolbachia* and *Rickettsia* in insect resistance development. Curr Opin Insect Sci. 2019;33:84–90. 10.1016/j.cois.2019.05.003. 31358201 10.1016/j.cois.2019.05.003

[72] Fan Z-Y, Liu Y, He Z-Q, Wen Q, Chen X-Y, Khan MM, et al. *Rickettsia* infection benefits its whitefly hosts by manipulating their nutrition and defense. Insects. 2022;13:1161. 10.3390/insects13121161. 36555070 10.3390/insects13121161 PMC9785894

[73] Hendry TA, Hunter MS, Baltrus DA. The facultative symbiont *Rickettsia* protects an invasive whitefly against entomopathogenic *Pseudomonas syringae* strains. Appl Environ Microbiol. 2014;80:7161–8. 10.1128/AEM.02447-14. 25217020 10.1128/AEM.02447-14 PMC4249164

[74] McCutcheon JP, Boyd BM, Dale C. The life of an insect endosymbiont from the cradle to the grave. Curr Biol. 2019;29:R485–95. 10.1016/j.cub.2019.03.032. 31163163 10.1016/j.cub.2019.03.032

[75] Bennett GM, Moran NA. Heritable symbiosis: The advantages and perils of an evolutionary rabbit hole. Proc Natl Acad Sci. 2015;112:10169. 10.1073/pnas.1421388112. 25713367 10.1073/pnas.1421388112 PMC4547261

