# Supplementary material for: Stable nutritional endosymbiosis across cryptic diversity of a leafhopper species complex
Source: BMC Genomics. 2026 May 25;27:624. doi: 10.1186/s12864-026-12986-3 (PMC13383532; doi:10.1186/s12864-026-12986-3)
Supplement: Supplementary file 1 — Supplementary Material 1. [file 12864_2026_12986_MOESM1_ESM.docx]

Stable nutritional endosymbiosis across cryptic diversity of a leafhopper species complex

Anna Michalik^1*^, Emilia Majewska^2^, Veronika Andriienko^1,3^, Karol H. Nowak^2,3^, Adam Stroiński^4^, Piotr Łukasik^2^

^1.^ Department of Developmental Biology and Invertebrate Morphology, Institute of Zoology and Biomedical Research, Faculty of Biology, Jagiellonian University, Kraków, Poland

^2.^ Institute of Environmental Sciences, Faculty of Biology, Jagiellonian University, Kraków, Poland

^3.^ Doctoral School of Exact and Natural Sciences, Jagiellonian University, Kraków, Poland

^4.^ Museum and Institute of Zoology, Polish Academy of Sciences, Warsaw, Poland

* Correspondence: a.michalik@uj.edu.pl

Supplementary Figures

**Figure S1.** Heatmap showing the distribution of the most abundant bacterial zOTUs in Verdanus populations

**Figure S2.** The relative abundance of dominant ITS1 zOTUs across individual leafhoppers from across 15 populations.

**Figure S3.** The relative abundance of dominant ITS2 zOTUs across individual leafhoppers from across 17 populations.

**Figure S4.** The relative abundance of dominant 18S rDNA zOTUs across individual leafhoppers from across 17 populations.

**
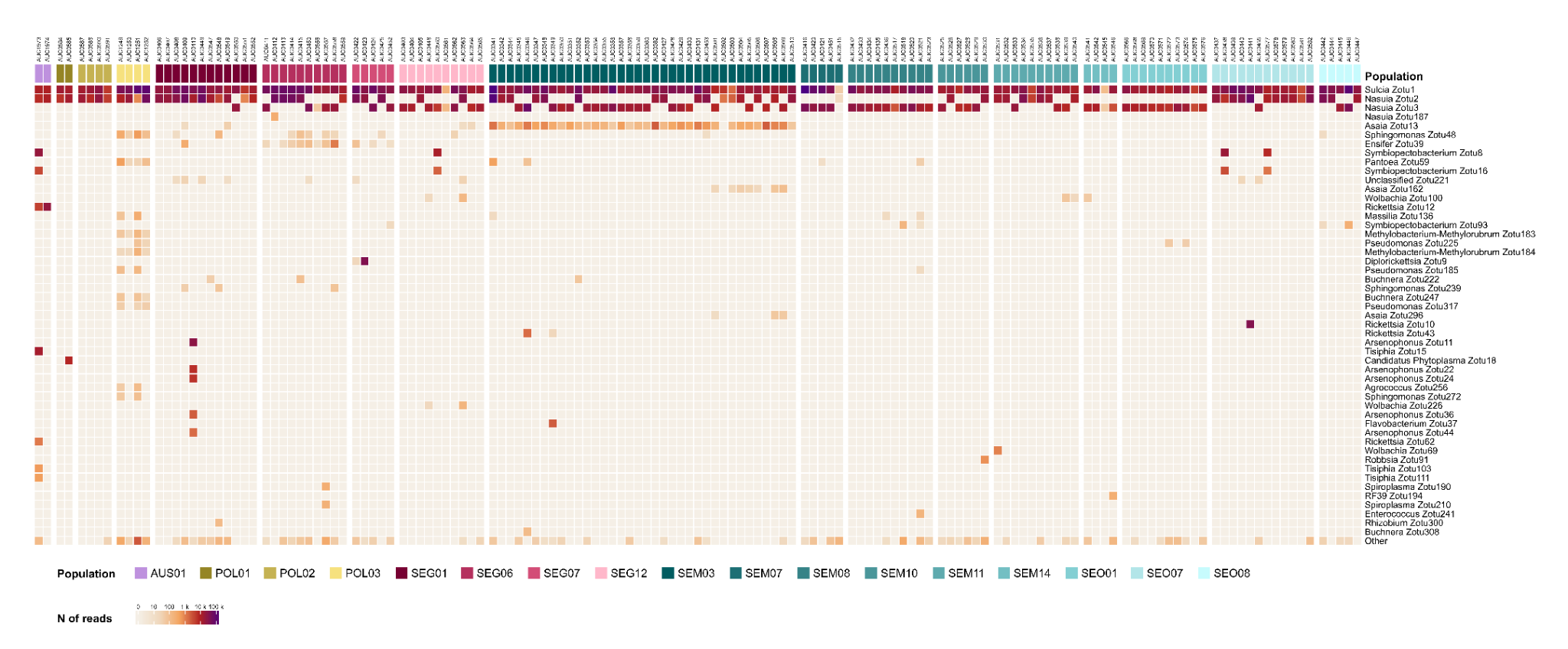
**

**Figure S1.** Heatmap showing the distribution of the most abundant bacterial zOTUs in the *Verdanus* populations.

**
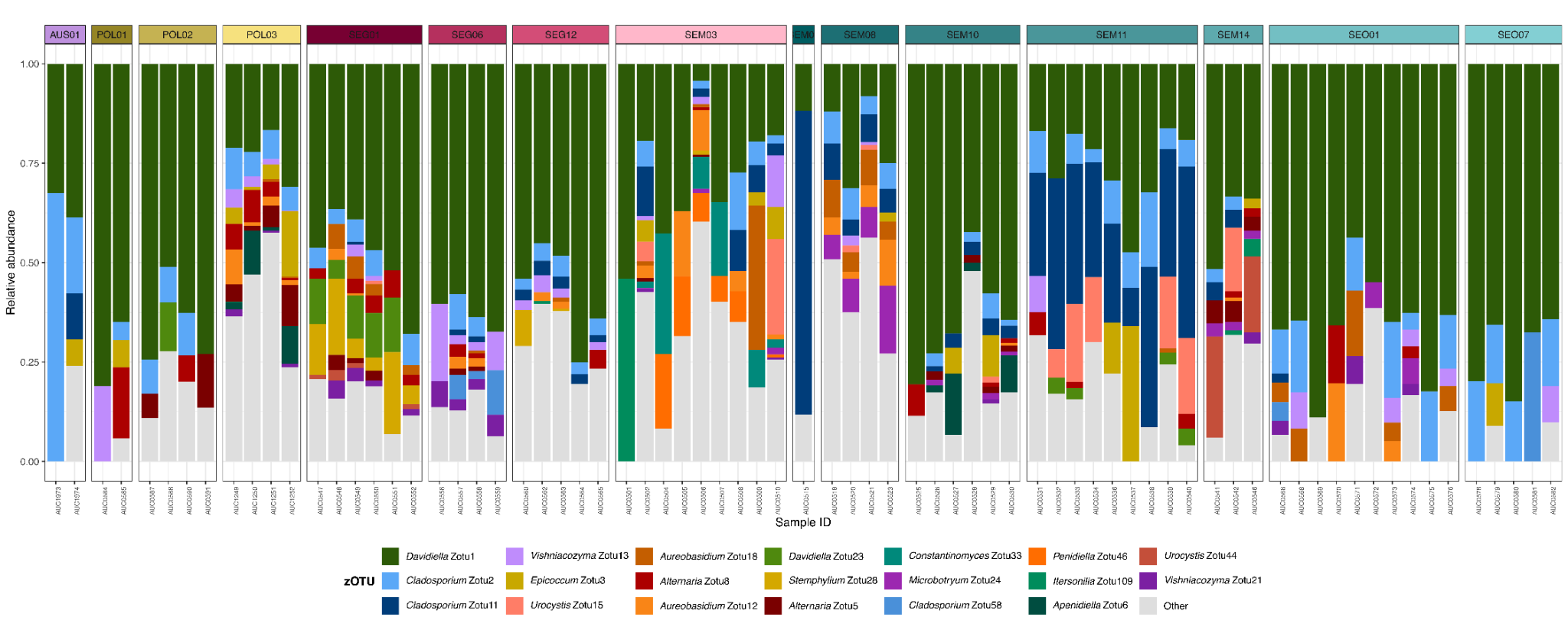
**

**Figure S2.** The relative abundance of dominant ITS1 zOTUs across individual leafhoppers from across 15 populations.

**
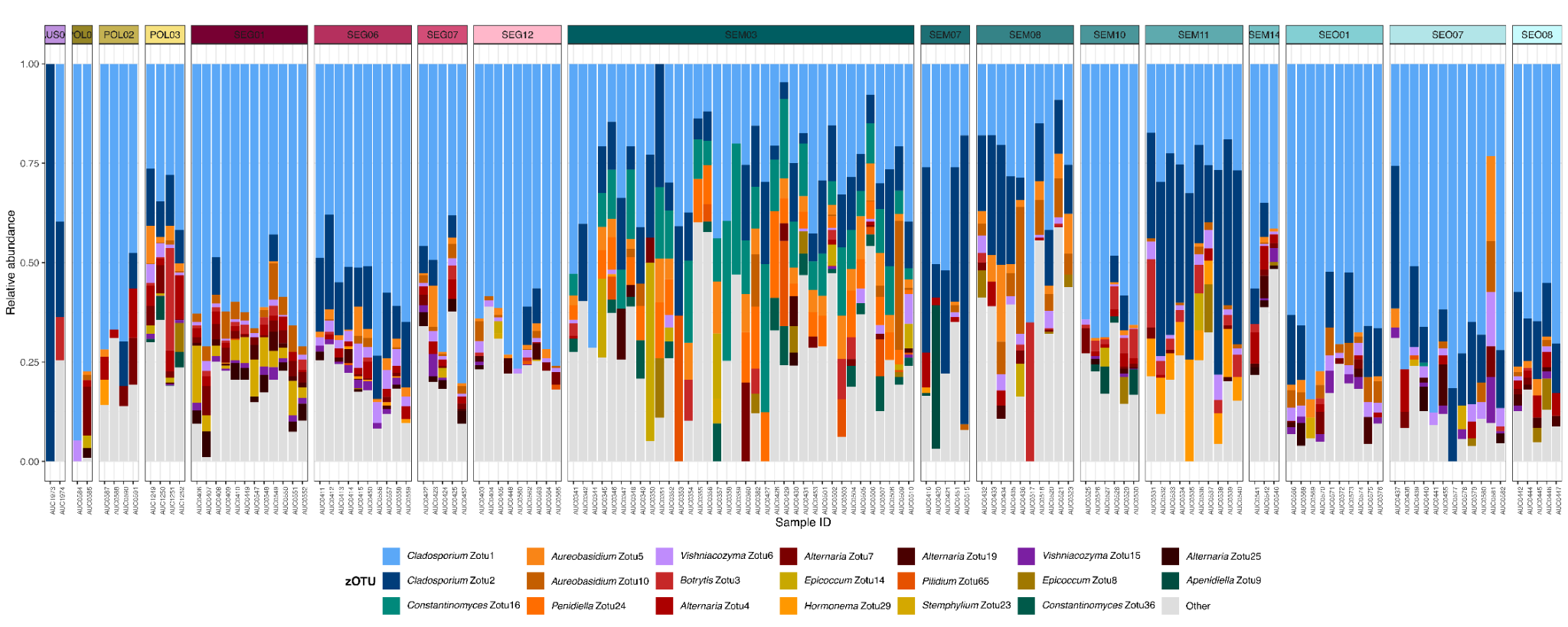
**

**Figure S3.** The relative abundance of dominant ITS2 zOTUs across individual leafhoppers from across 17 populations.

**
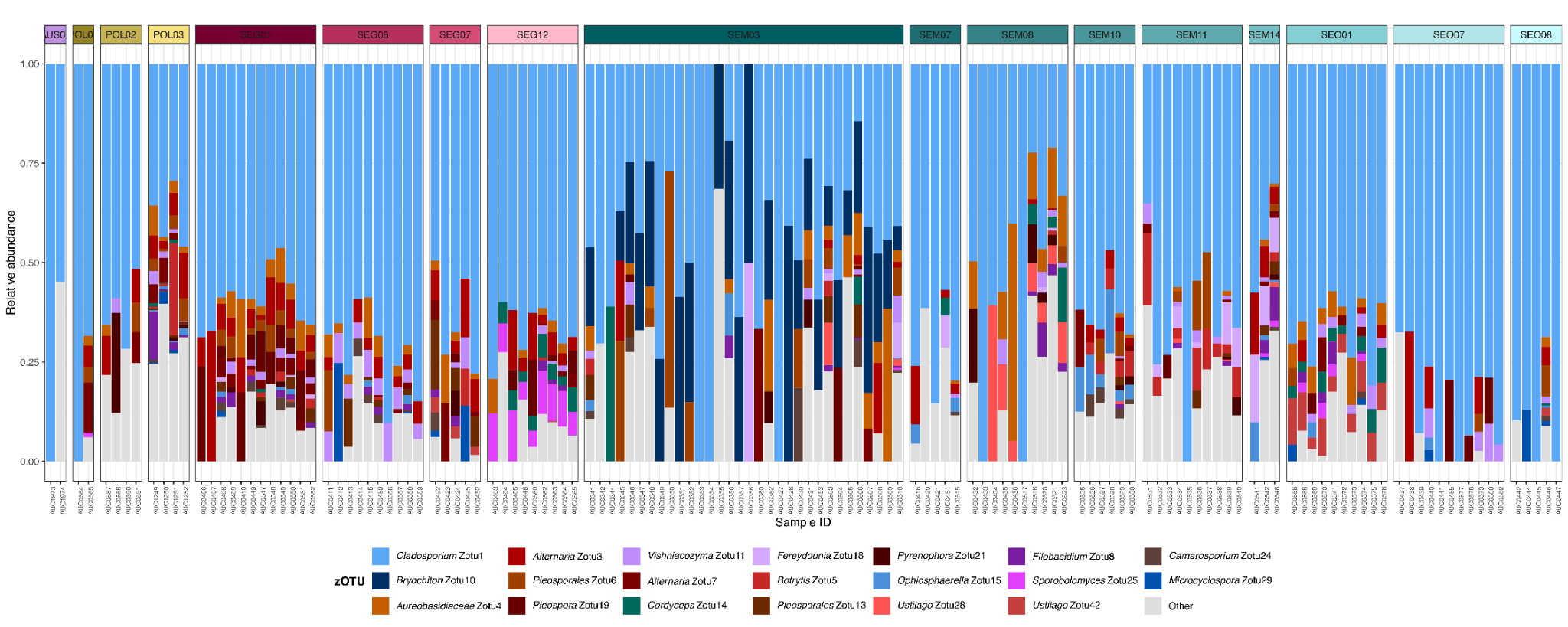
**

**Figure S4.** The relative abundance of dominant 18S rDNA zOTUs across individual leafhoppers from across 17 populations.
